# Embolization-induced Renal Tumor Shrinkage Followed by Definitive Cryoablation

**DOI:** 10.7759/cureus.3251

**Published:** 2018-09-04

**Authors:** Jerry Matteo, Todd Loper, Preston Hood, Erik Soule, Joanna Kee-Sampson, Jesse T Martin

**Affiliations:** 1 Interventional Radiology, University of Florida College of Medicine, Jacksonville, USA; 2 Interventional Radiology, Flagler Hospital, St. Augustine, USA; 3 Interventional Radiology, University of Florida Health, Jacksonville, USA; 4 Radiology, University of Florida Shands-Jacksonville, Jacksonville, USA; 5 Medical Student, Edward Via College of Osteopathic Medicine, Auburn, USA

**Keywords:** cryoablation, embolization, embolization prior to cryoablation

## Abstract

Significant incidental findings reported on computed tomography (CT) scans are common. This article describes a 72-year-old man evaluated for possible bowel obstruction in whom was found a 3.1-cm x 2.6-cm centrally located enhancing mass in the left kidney highly suspicious for renal cell carcinoma. Due to substantial medical comorbidities, the patient was deemed a poor surgical candidate for either partial or complete nephrectomy. Interventional radiology was consulted for a minimally invasive ablation procedure. The large size and central location of the tumor involving the renal collecting system initially precluded definitive percutaneous cryoablation. Intra-arterial embolization was used as neoadjuvant therapy to decrease tumor burden. Fluoroscopy-guided bland embolization was performed targeting the arterial supply of the mass until stagnation of flow was achieved. A subsequent two-month post-embolization follow-up CT scan showed a 30% reduction in tumor size. Shrinkage of the mass from a central to a more peripheral location allowed for a cryoablation approach that would avoid damage to the vulnerable collecting system. Cryoablation was performed, and intraoperative CT demonstrated complete coverage of the tumor by the ice ball with no damage to the renal collecting system. A follow-up CT scan four years later showed no residual malignancy at the ablation site.

## Introduction

The incidence of renal masses has increased significantly in recent years due to the increase in axial imaging performed for unrelated reasons [1]. Renal cell carcinoma is the most common type of malignant tumor found in the kidneys, accounting for approximately 85% of cases [2]. The gold standard for treating such solitary malignancies is partial or complete nephrectomy. The problem many patients face with partial or complete nephrectomy is the morbidity and mortality associated with such an invasive procedure as well as a significant loss in functioning of renal parenchyma, especially in patients with poor or borderline renal function at baseline. Cryoablation provides an excellent and potentially curative option to those who are poor surgical candidates or to those in whom the loss in functional renal parenchyma cannot be afforded. Cryoablation is a percutaneous procedure performed under ultrasound or computed tomography (CT) guidance that allows the operator to precisely freeze a tumor into a lethal ice ball which results in tumor cell death. The procedure is minimally invasive and allows for shortened post-procedure hospitalization and recovery time compared to surgery. The cure rates for nonmetastatic (i.e., stage 1A or 1B) renal cancers treated with cryoablation are between 90% and 97% at five years [3].

Bland arterial embolization is currently used to debulk tumors such as uterine fibroids as well as hyperplastic tissue such as that found in benign prostatic hyperplasia [4-5]. This is accomplished using fluoroscopy by selectively embolizing the feeding vessels of these structures which results in ischemia followed by shrinkage. Arterial embolization in the treatment of cancer is generally not effective on its own in producing long-term remission or cures due to the rapid formation of collaterals [6]. Renal tumor arterial embolization has been used commonly prior to planned surgical resection to limit intraoperative or post-procedural bleeding with success [7]. The use of embolization to reduce tumor burden prior to cryoablation to limit potential damage to the renal collecting system during treatment of central renal tumors has not been documented. Although cryoablation and arterial embolization are commonly used in the treatment of renal cell carcinoma, the successive combination to shrink renal tumors prior to embolization has not been documented. A literature search using the PubMed electronic database yielded no cases similar to the case presented in this article.

## Case presentation

**Figure 1:**
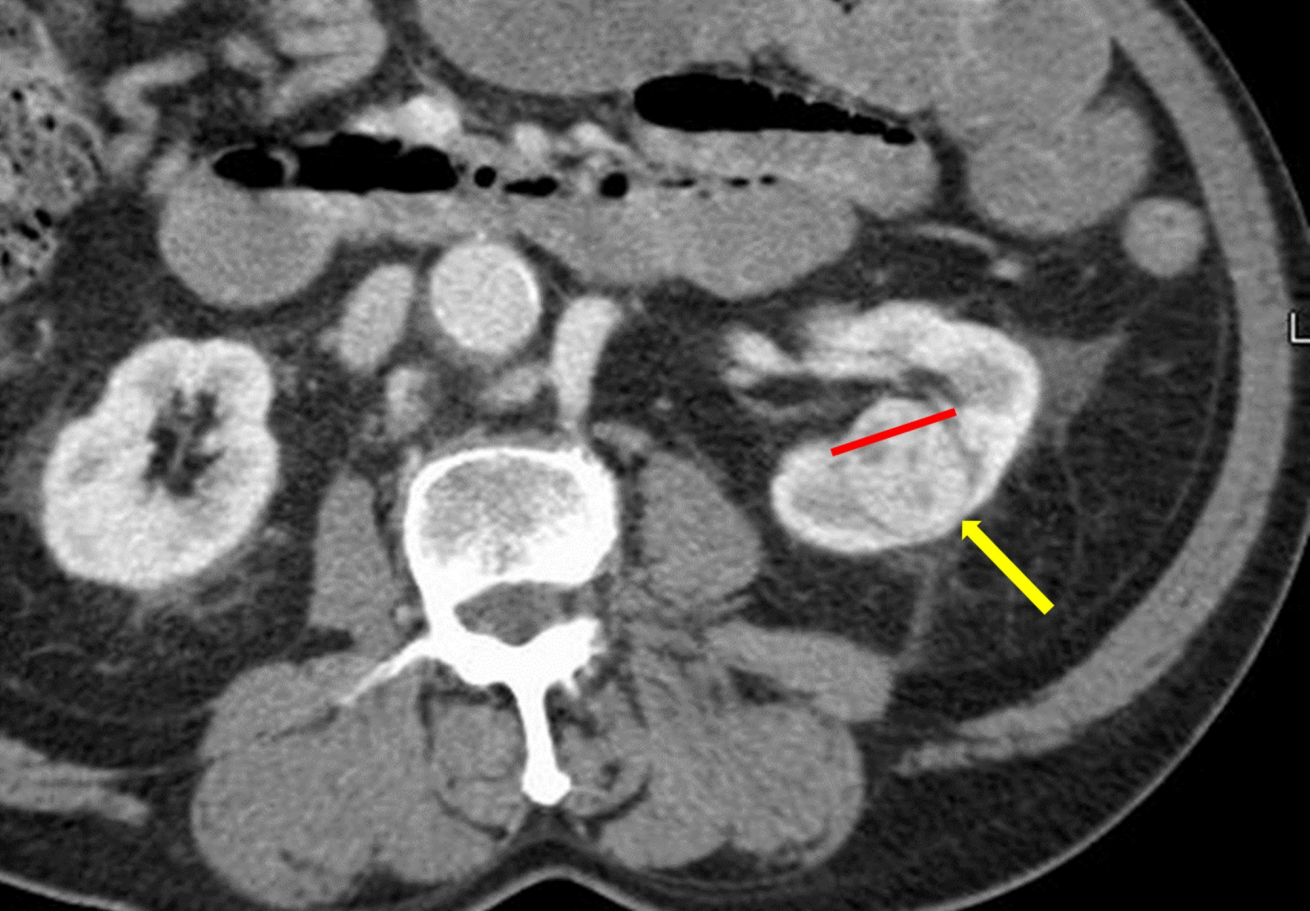
Initial coronal CT showing an incidental renal mass measuring 3.1 cm x 2.6 cm (yellow arrow) with extension into the renal pelvis (red line). Abbreviation: CT, computed tomography.

**Figure 2:**
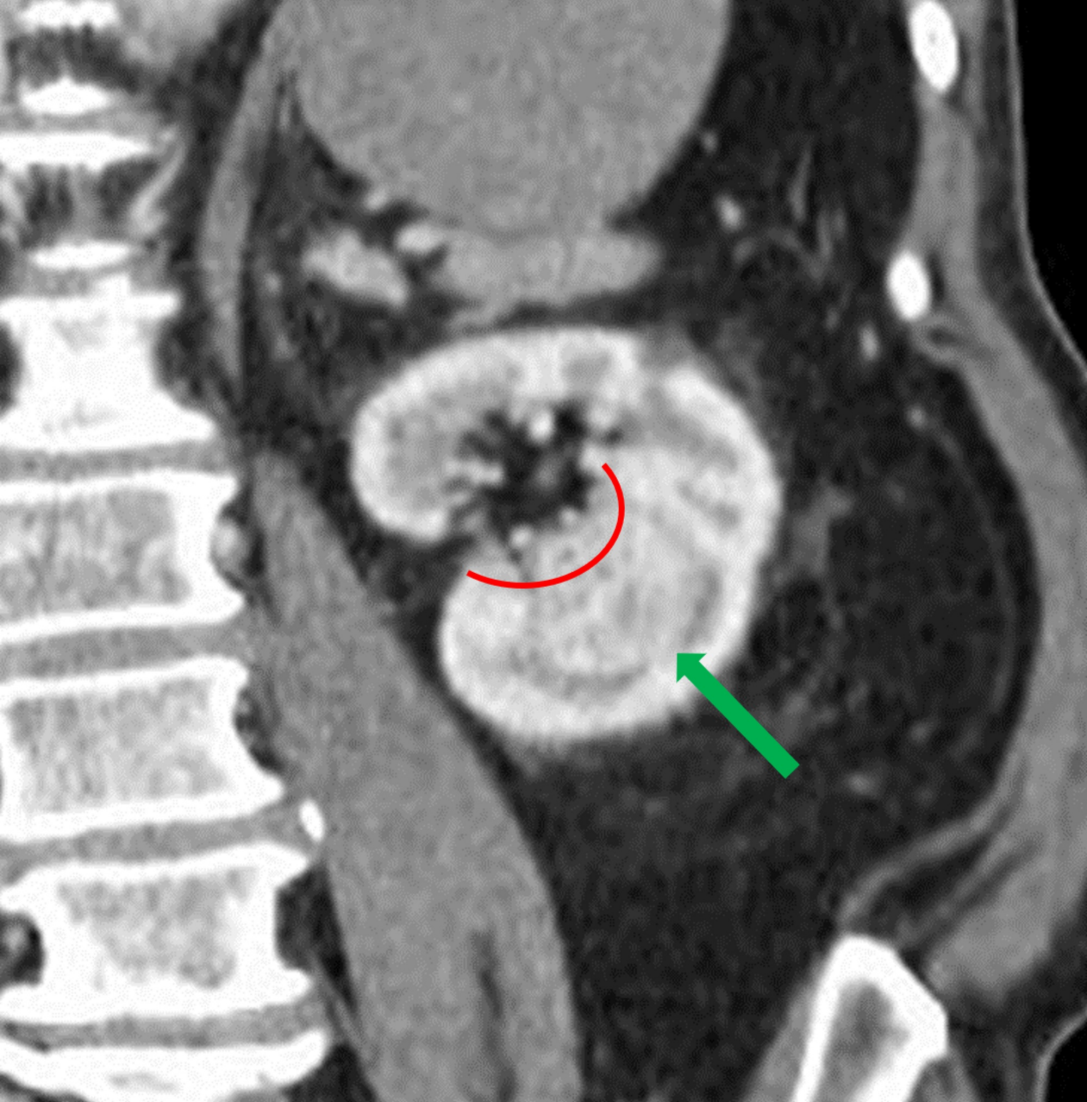
Initial coronal CT showing an incidental renal mass measuring 3.1 cm x 2.6 cm (green arrow) with extension into the renal pelvis (red curved line). Abbreviation: CT, computed tomography.

A 72-year-old man with a past medical history of abdominal aortic aneurysm repair, prostate cancer, ischemic stroke with residual left hemiparesis, and significant peripheral vascular disease complicated with an above-the-knee amputation presented to our departments following an abdominal CT for possible bowel obstruction with a 3.1-cm x 2.6-cm solid renal tumor (Figure 1). The renal tumor was enhancing and located centrally at the inferior pole of the left kidney (Figure 2).

This patient was referred to interventional radiology because he was deemed a poor surgical candidate due to his underlying comorbidities. Due to the central location and the high probability of irreversible cryoablation of the renal collecting system, the decision was made to proceed with bland arterial embolization. This was done with the expectation that the tumor would shrink enough to allow a safe window for cryoablation.

**Figure 3:**
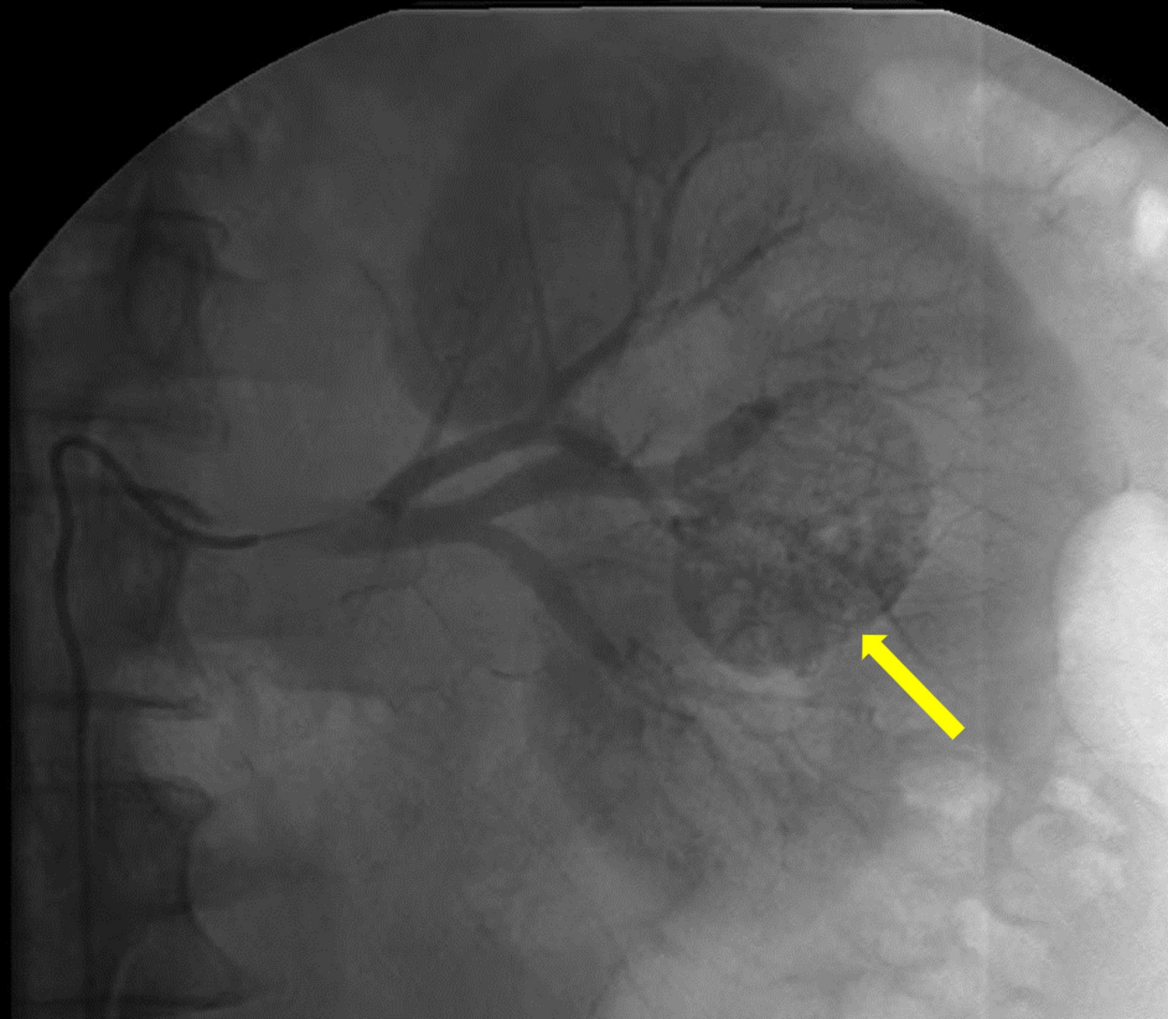
Angiography demonstrating areas of vascular supply of the renal mass (yellow arrow).

**Figure 4:**
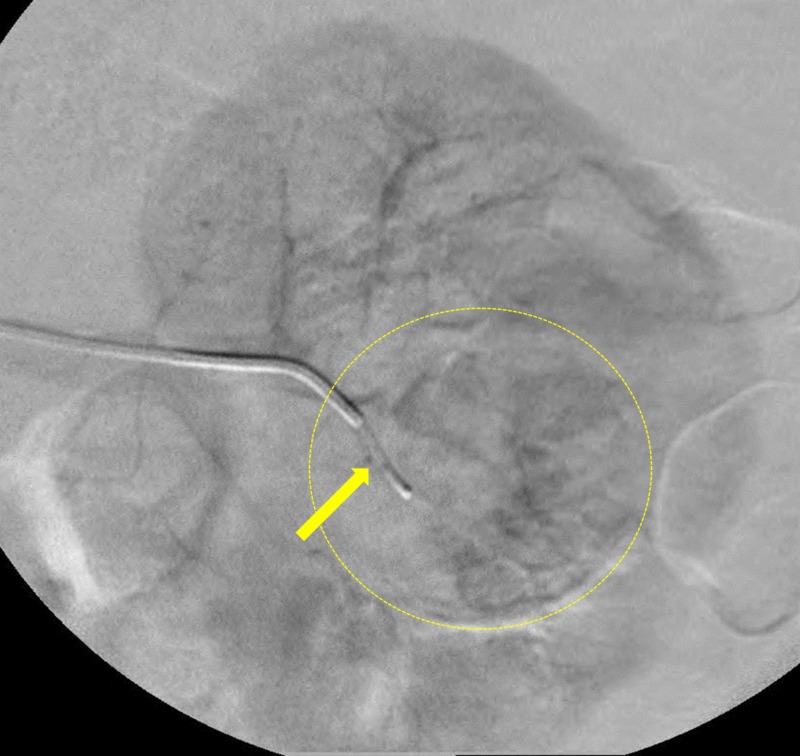
Selective catherization using a microcatheter (yellow arrow) of the feeding vessels supplying the tumor (yellow dashed circle).

**Figure 5:**
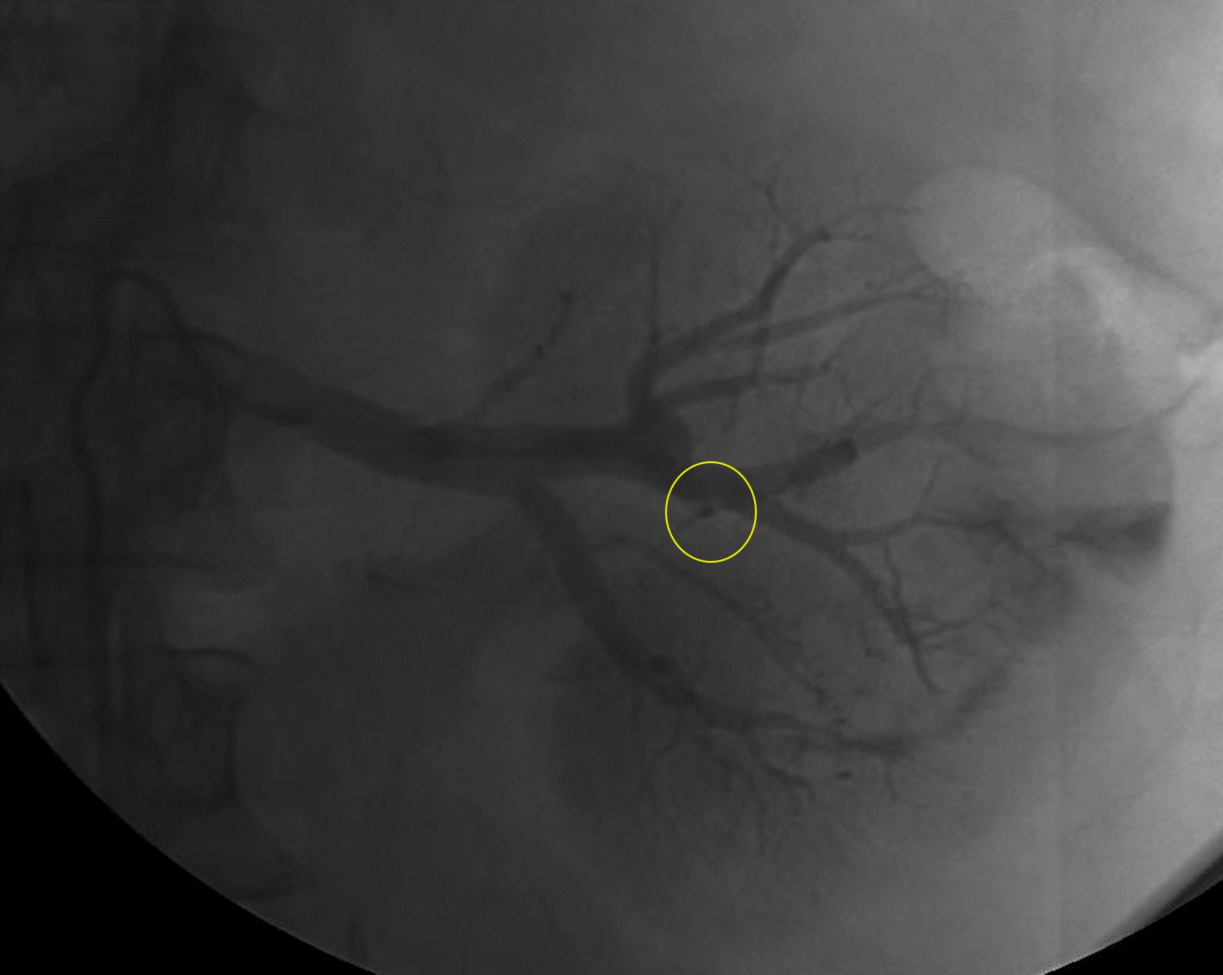
Post-embolization angiography demonstrating cessation of vascular flow in the main arterial supply to the tumor (yellow circle) with loss of vascular supply to the tumor indicating adequate bland embolization.

**Figure 6:**
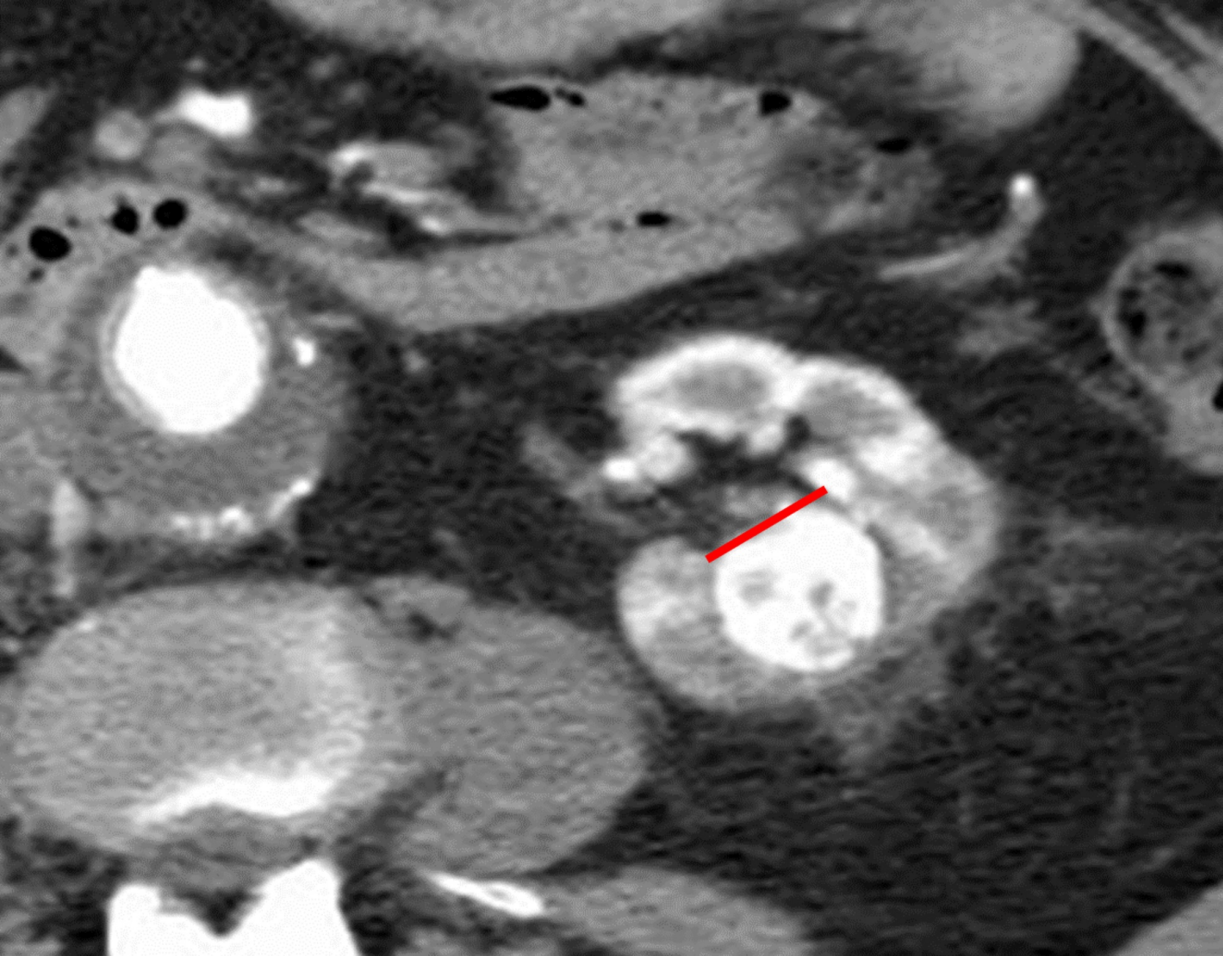
Follow-up axial CT two months post-embolization showing a 30% reduction in tumor size with loss of tumor extension into the renal pelvis (red line). Abbreviation: CT, computed tomography.

Conventional angiography was performed which demonstrated enhancement of a well-defined vascular, renal mass (Figure 3). Super selective catheterization using a microcatheter of the feeding vessels allowed for selective bland embolization of the tumor with 100-300 microns polyvinyl alcohol particles (Figure 4). Post-embolization angiography showed a cessation of vascular flow to the tumor (Figure 5). Follow-up CT two months post-embolization showed a 30% reduction in tumor size (Figure 6).

**Figure 7:**
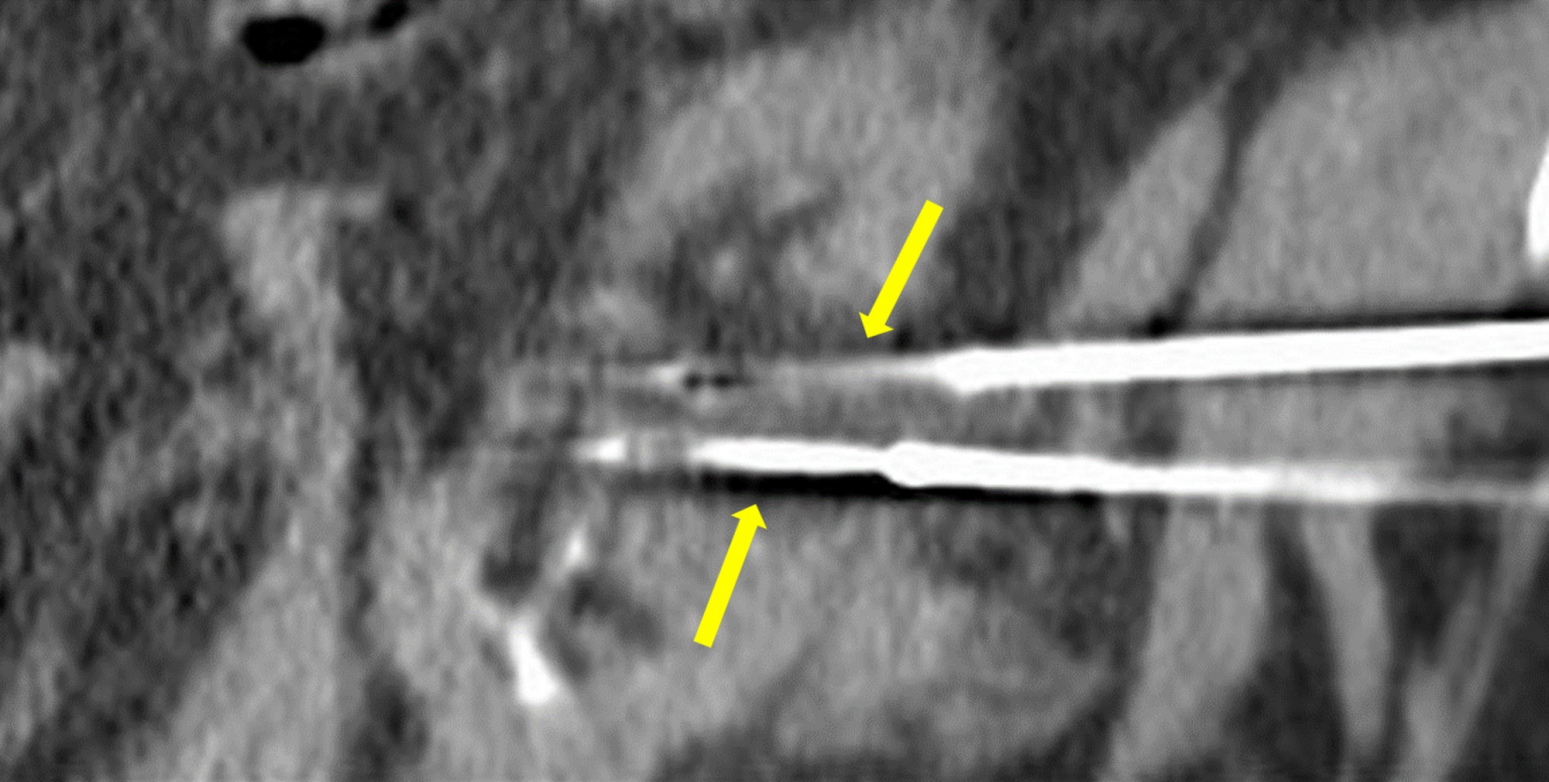
Pre-cryoablation cryoprobes positioned in the center of the tumor (yellow arrows).

**Figure 8:**
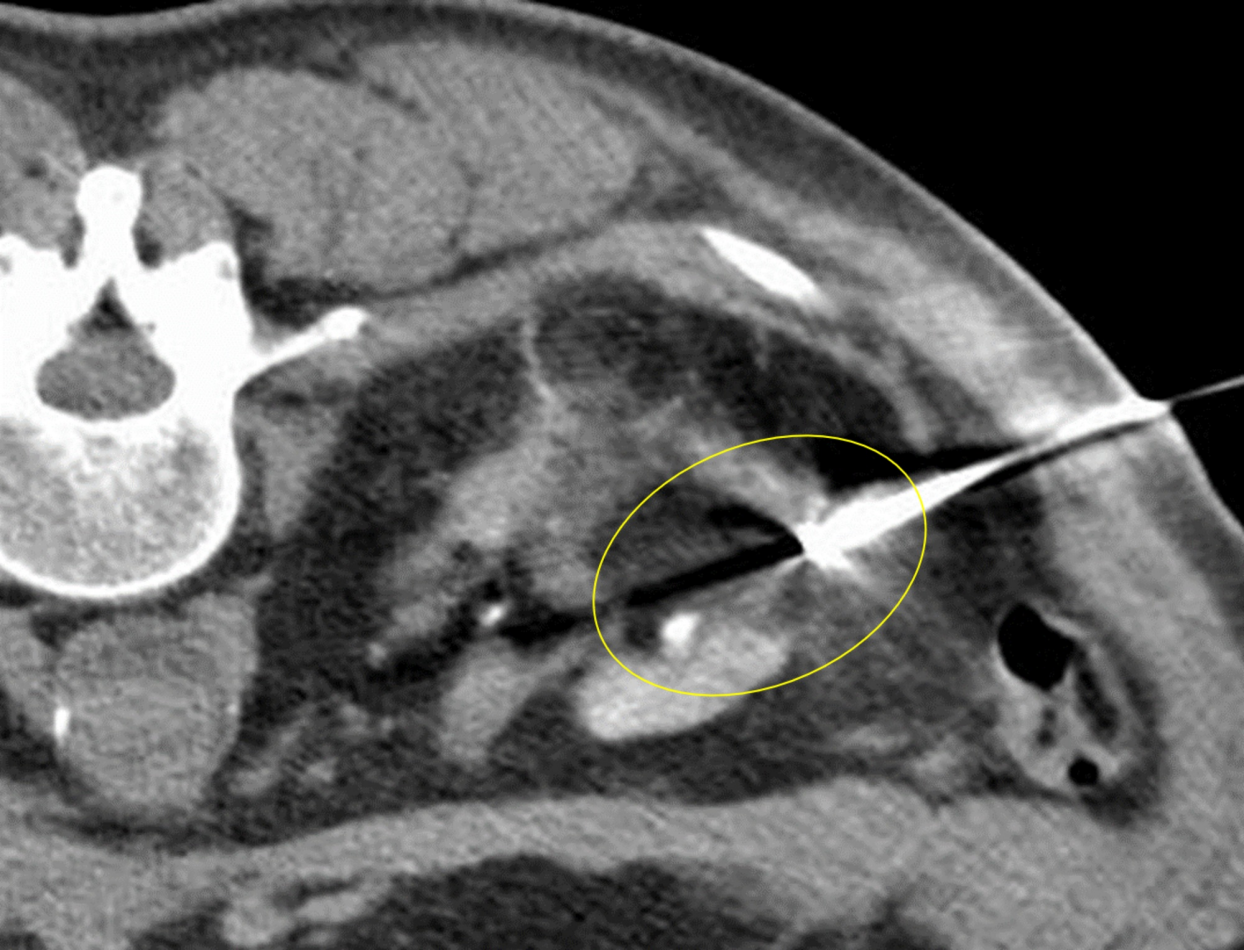
Hypoattenuated area engulfing the tumor representing formation of the ice ball tumor (yellow circle).

**Figure 9:**
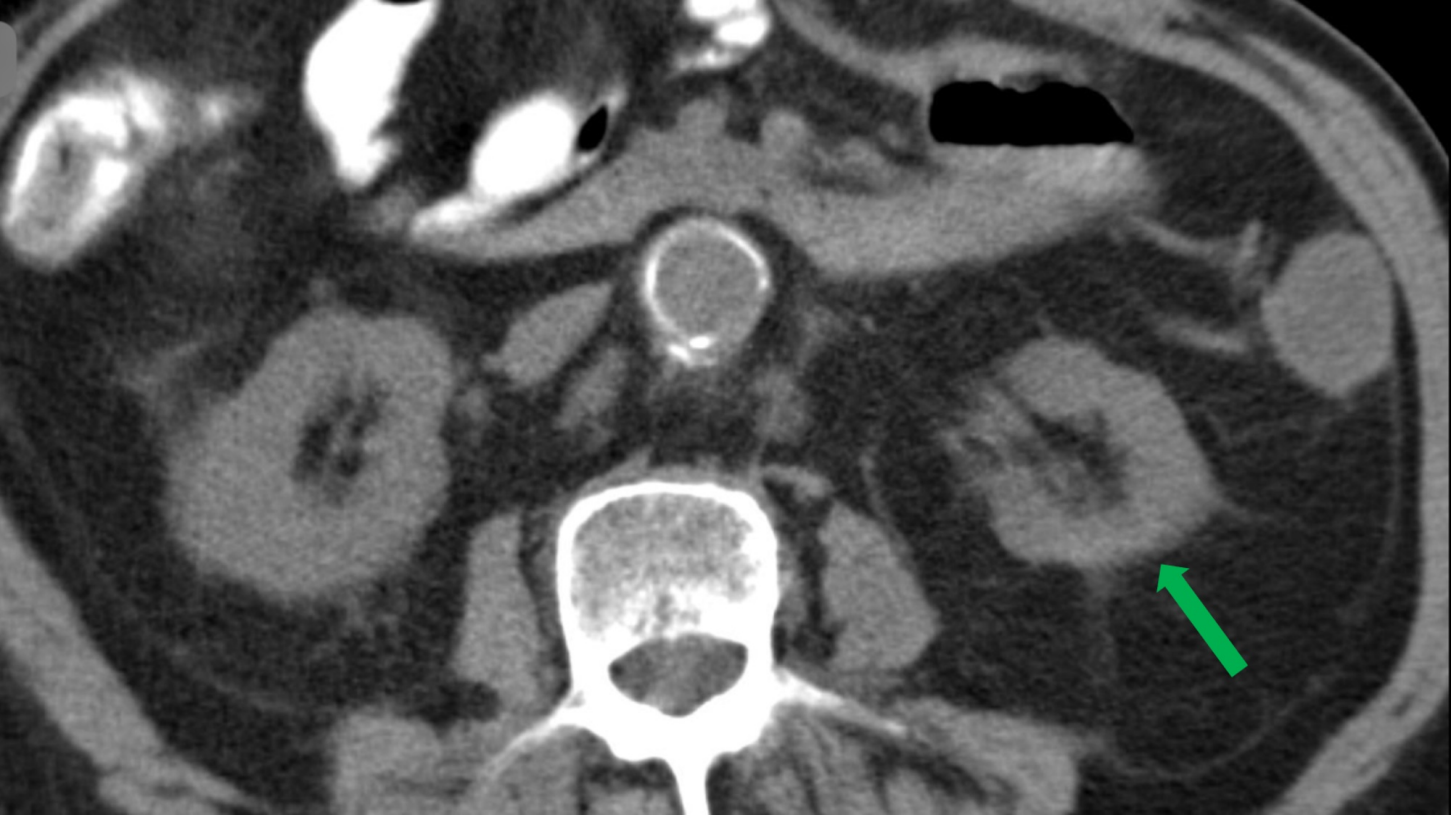
Four-year post-embolization follow-up axial CT showing no residual tumor in the left kidney (green arrow). Abbreviation: CT, computed tomography.

Three months following initial embolization, the patient underwent cryoablation of the renal tumor. Two cryoprobes were inserted into the center of the tumor under CT guidance (Figure 7), and two freeze cycles were performed (10 minutes each) interspersed by a thaw cycle of eight minutes. Intraoperative images demonstrated complete coverage of the residual tumor by the ice ball (Figure 8). The patient tolerated the procedure well and had no immediate post-procedure complications. The renal collecting system was not affected by the cryoablation procedure. A follow-up CT four years after the cryoablation showed no residual malignancy (Figure 9).

## Discussion

With the widespread use of CT, ultrasound, and magnetic resonance imaging, the incidence of diagnosed renal masses will likely continue to rise. In multiple studies, the five-year survival and cure rates for cryoablation are shown to be greater than 90%, making it more attractive as a first-line therapy for a large population of patients found to have these tumors. Many incidental renal tumors are exophytic, meaning they are in the renal cortex extending away from the inner renal parenchyma [8]. These tumors are amenable to direct cryoablation because the inner renal parenchyma and collecting system is less likely to sustain cryoablative insult [9]. When using pre-adjuvant bland embolization such as the one described here, allowing several months between embolization and cryoablation may be preferable. Performing cryoablation several months post-embolization allows for adequate tumor shrinkage, as well as recovery of renal function [10]. Similarly, to achieve adequate post-bland embolization shrinkage for other tumors such as uterine fibroids, two months between procedures is usually adequate to achieve the desired size reduction [4].

In renal tumors that are large or extend centrally, novel approaches such as that described here may allow cryoablation to be safely performed. This case report describes bland embolization induced tumor shrinkage as a pre-adjuvant interventional therapy to cryoablation. For many years, the use of preoperative chemotherapy to reduce tumor burden in various cancers prior to surgery has been employed with success [11]. Analogous to presurgical chemotherapy, as cryoablation becomes the preferred treatment for renal carcinoma, precryoablative bland embolization may allow for treatment of previously inaccessible tumors. This technique may be applicable to kidney tumors and possibly other malignancies as well.

## Conclusions

The majority of renal tumors are exophytic and are amenable to definitive percutaneous cryoablation. This case represents a novel two-part approach to the treatment of large, centrally located renal tumors. Pre-adjuvant bland embolization was employed with subsequent cryoablation. This allowed the operator to minimize the risk of damage to the surrounding renal parenchyma and collecting system during cryoablation. The method described offers a safe and effective treatment modality for a difficult renal tumor location.
